# Selected drugs that inhibit DNA methylation can preferentially kill p53 deficient cells

**DOI:** 10.18632/oncotarget.2441

**Published:** 2014-09-10

**Authors:** Lan Yi, Yvonne Sun, Arnold Levine

**Affiliations:** ^1^ Rugters Cancer Institute of New Jersey, New Brunswick, New Jersey; ^2^ Department of Pediatrics, Rutgers New Jersey Medical School, Piscataway, New Jersey; ^3^ The Simons Center for Systems Biology, Institute for Advanced Study, Princeton, New Jersey

**Keywords:** FCDR, Decitabine, Zebularine, EGCG, RG108, DNA methylation inhibitor, p53

## Abstract

The p53 protein ensures cellular fidelity by suppressing or killing cells under stresses that enhance the mutation rate. Evidence suggests that the p53 protein may also ensure the fidelity of the epigenome. In this study a group of drugs that alter the deoxycytosine methylation patterns in cellular DNA are shown to preferentially kill human and mouse cells that contain p53 mutations or deficiencies. These observations are extended to mice that contain p53 deficiencies or missense mutations in their genome, which are preferentially killed when compared to mice with a wild type p53 gene. This is also the case for human cancer cell xenografts containing p53 mutations, which preferentially are killed by these drugs when compared to similar tumors with wild type p53. The loss of p53 function enhances a synthetic lethality with drugs that block or alter the patterns of deoxycytidine methylation in the genome.

## INTRODUCTION

The p53 protein is a transcription factor that responds to a wide variety of stresses. These stresses include DNA damage, metabolic alterations in a cell, hypoxia, the interruption of ribosomal biogenesis, oncogene activation and even viral infections. There are several types of p53- mediated responses to these stress signals, which include DNA repair, metabolic changes, cell cycle arrest, cellular senescence and apoptosis that may mediate tumor suppression. Because these types of stress occurring during cell division can enhance the mutation rates in cells, p53 enforces the fidelity of the division cycle lowering the rate of tumorigenesis.

Recently several lines of evidence have suggested that the p53 protein may also prevent epigenetic changes from occurring during division or development. Experiments by Jackson-Grusby et al [1] demonstrated that the loss of the DNA-methyl-transferase-1 gene from cells in culture, resulted in the failure to copy methyl-cytosine residues in the DNA and after two cell divisions the cells died of a p53-mediated apoptosis. Employing a quite different approach, Yamanaka and colleagues [2] demonstrated that it was possible to reprogram fibroblasts into induced pluripotent stem cells (IPS cells) by employing four transcription factors, myc, Klf4, Sox-2 and Oct-4. This process is inefficient (about 0.1% of the cells form IPS cells) and takes a long time in culture. In the absence of p53 however only Sox-2 and Oct -4 will produce IPS cells at much higher efficiencies (up to 80%) and much shorter times [3–8]. This suggests that wild type p53 slows the rate of epigenetic reprograming [9] in both normal cells and cancer cells. Indeed the wild type p53 protein is inactive in embryonal carcinoma cells (EC cells), the stem cell of testicular cancers [10] and IPS cells will differentiate when wild type p53 is activated or introduced [9]. In human breast cancer cells [11] there is a strong correlation between the loss of p53 functions and the presence of an embryonic stem cell mRNA profile or signature as observed by microarrays. In many cancer cells the loss of p53 function permits the transcription of repetitive DNA elements via epigenetic changes in the repetitive DNA sequences and chromatin [12, 13]. In Planaria, the p53 protein regulates both proliferation and stem cell renewal for regeneration [14] and in the salamander, p53 controls cell plasticity in limb regeneration [15]. Indeed the earliest phenotype of p53 function was cellular self-renewal or immortalization, which was highly correlated with p53 mutation in cell culture[16]. Taken together these observations suggest that p53 functions monitor or regulate stem cell states and epigenetic changes that produce stem cells or progenitor cells.

DNA methylation is one of the major epigenetic markers for gene repression. Many reports have demonstrated a strong association between disruption of DNA methylation and the formation of neoplasia [17]. In cancer cells promoter hypermethylation of CpG islands occurs frequently to silence many tumor suppressor genes. On the other hand, the total DNA is generally hypomethylated in tumors commonly due to the undermethylation of repetitive elements in heterochromatic regions of the genome [17]. A number of chemical DNA methylation inhibitors have been developed and have been classified into nucleoside analogues and non-nucleoside analogues. The nucleoside analogues are incorporated into the DNA or RNA by replication or transcription. For example cytosine analogues 5-aza-cytotidine (Vidaza) and 5-aza-2-deoxycytidine (Decitibine) were shown to reactivate many aberrantly repressed genes in tumors inducing anti-proliferative activity. The non-nucleotide cytosine methylation inhibitors act by inhibiting the DNA methyl-transferases at their catalytic site [18].

In 2004, Nieto et al [19] first reported that 5-aza-2-deoxycytidine selectively killed p53 deficient mouse embryo fibroblasts (MEFs) but failed to kill MEFs that contained wild type p53 protein. Mutations in other p53 related pathway proteins such as p19-ARF, P16 INK4a, p21, E2F-1 or E2F 2 all failed, unlike p53 mutants, to permit killing of the cells by this drug. These results were followed up by Leonova et al [13] who provided a mechanism for this observation. She and her colleagues demonstrated that in the absence of wild type p53 epigenetic changes in the genome lead to the expression of repetitive DNA sequences and the resultant RNAs (some of which form extensive secondary structures) induced interferon production resulting in cell death (i.e. activation of the innate immune response).

In this publication we confirmed and extended these observations. In addition to 5-aza-2-deoxycytidine, we found a number of DNA methylation inhibitors could induce greater levels of apoptosis in p53 deficient MEFs than in wild type cells both in cell culture and in vivo. These results were extended to live mouse models and to human xenograft cancer models. These data contribute additional evidence that the p53 protein plays a critical role in epigenetic regulation that has important consequences for cancer treatments.

## RESULTS

### P53 deficient or mutant mouse embryonic fibroblasts (MEF) were hypersensitive to two types of DNA methylation inhibitors

**Figure 1:**
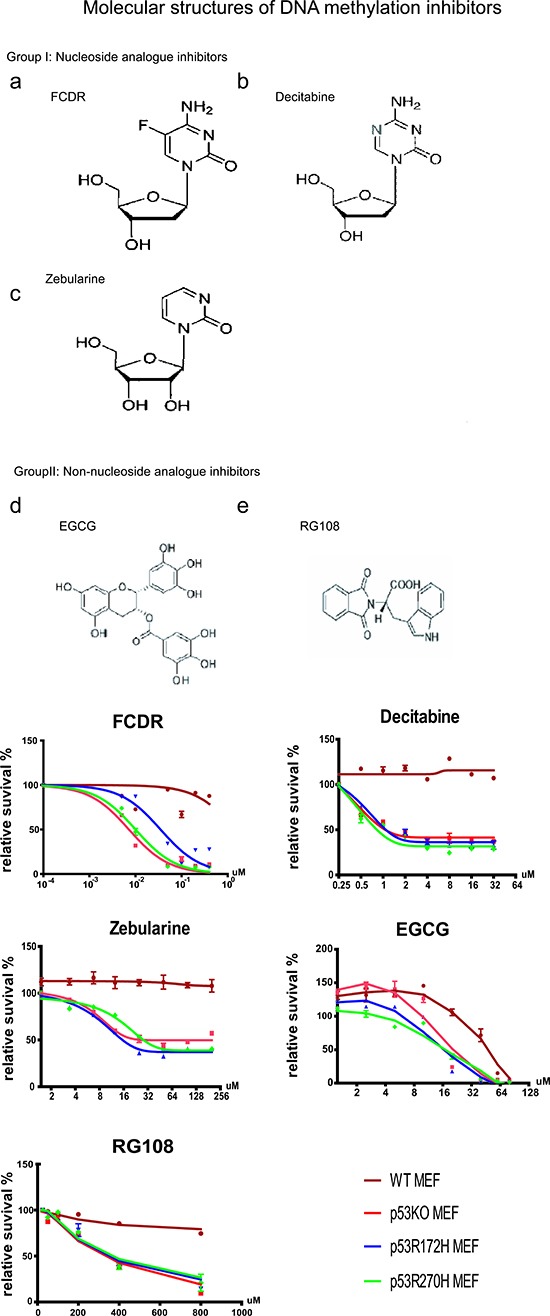
Cell Viablility Assay for five demethylation drugs in MEFs **(A)** Molecular structures of five DNA methylation inhibitors. Nucleoside analogues (a-c) and Non-nucleoside analogues (d,e). **(B)** Cell relative survival plot by Guava assay of 5 demethylation agents in primary MEF cells (P0-P2). FCDR (5-fluoro-20-deoxycytidine); Decitabine (5-aza-2-deoxycytidine); Zebularine (1-beta-D-ribofluranosyl-2(1H)-pyrimidinone); EGCG (-)-epigarllocatechin-3-gallate; RG108 (N-Phthalyl-L-Tryptophan); P53wt (brown, cycle); p53KO(red, square); p53R172H(blue, triangle) & p53R270H(green, diamond).

Two previous publications reported that the DNA demethylation agent Decitabine unexpectedly induced more apoptosis in p53 null or mutated cells than in p53 wildtype cells [13, 19]. These results posed the hypothesis that partial inhibition of DNA methylation may cause lethality for p53 deficient cells but not for wildtype cells. To examine this hypothesis and explore more potential drugs, we applied guava cell viability assays to 5 different DNA demethylation reagents, FCDR, Decitabine, Zebularine, EGCG, RG108. Three of them were nucleoside analogues. They were Decitabine, FCDR and Zebularine (Fig1Aa-c). Two were non-nucleoside analogue inhibitors, EGCG and RG108 (Fig1Ad,e). Their molecular structures are shown in Figure 1A. In our assay, four genotypes of MEFs were used including wildtype, p53 knock-out, p53 gain of function mutation r172h and r270h. p53r172h mutation is a structural mutation in the protein DNA binding domain, which led to a protein conformation change and loss of DNA binding capability of p53 [20]. The p53r270h mutation is a contact function mutation that occurs in the DNA binding domain which also affected p53 protein binding to DNA [18]. Both mutations commonly accumulate to high levels in tumor cells and are defective in wild-type p53 functions. To ensure that each cell line had a comparable high starting proliferation rate and avoid the possible artificial effect caused by long time cell culture, all the MEF cells used in this assay were at low passages (P0-P2). Serial dilution of drugs was given to each cell culture from day 0 to day 5, and the guava assays were accomplished at day 5.

As shown in figure 1B, after 5 drug treatments, all p53 deficient cells (p53KO, r172h or r270h) displayed very similar growth inhibition curves, clearly separating themselves from those for wild type cells. All three nucleoside analogue inhibitors were capable of killing p53KO/mut MEFs specifically. Among them, FCDR showed the highest efficiency in killing, while Zebularine showed the lowest efficiency. Non-nucleoside analogue inhibitor, EGCG, showed toxicity to both wild type and p53 deficient cells at higher dose, however, in the range from 10μM to 60μM, the wild type cells still showed more resistance to apoptosis than p53 deficient cells. Another non-nucleoside analogue inhibitor, RG108, on the contrary, showed very low toxicity to both wild type and p53 mutated cells, however, when the drug concentration was increased to above 100uM, it started to induce more cell death but only in p53 mutant cells.

### FCDR and Decitabine treatment did not immediately stop wild type cells from proliferation

**Figure 2:**
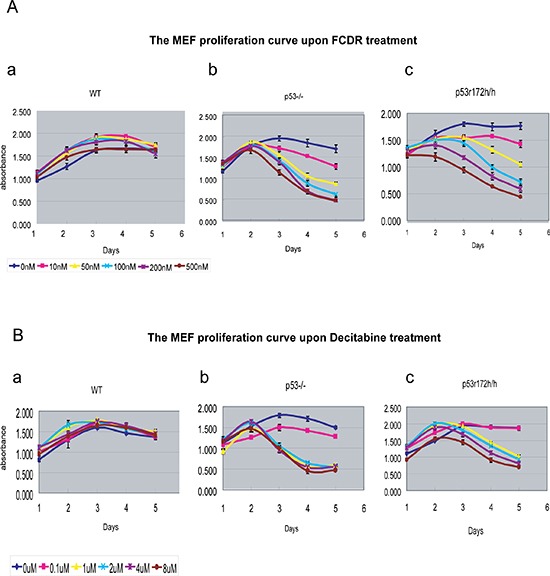
The MEF cell proliferation plots with 5 day time-trace and to gradient dosages of FCDR and Decitabine (**A**) The live cell proliferation curve with time upon series dosage of FCDR treatment in wildtype(a), p53KO(b) and p53r172h(c) MEFs. 0nM(dark blue), 10nM(light purple), 50nM(yellow), 100nM(light blue), 200nM(dark purple), 500nM(brown). (**B**) The live cell proliferation curve with time upon series dosage of decitabine treatment in wildtype(a), p53KO(b) and p53r172h(c) MEFs. 0μM(dark blue), 0.1μM(light purple), 1μM(yellow), 2μM(light blue), 4μM(dark purple), 8μM(brown).

Due to the existence of the wild type P53 protein in wild type MEFs, one can hypothesize that drug treatment could activate wild type p53 functions which might turn on the cell cycle arrest genes and stop cell from replication, therefore preventing them from cell death. To investigate this possibility, an assay over five days was carried out to follow the cell proliferation rate in primary MEF cells, treated with increasing levels of FCDR and Decitabine. As shown in Fig 2Aa & Ba, all wild type MEFs displayed similarly increasing growth curves from day1 to day 5. This proliferation did not change with increased doses of FCDR (Fig 2Aa) or Decitabine (Fig 2Ba). In contrast, p53 knock out MEFs showed clearly slower growth curves with increasing dosage of FCDR (Fig 2Ab) and Decitabine (Fig 2Bb). The proliferation rate significantly dropped with time. In addition, the slowest growth rates were strongly associated with the higher drug dosage. Similar results were also seen in p53 missense mutated r172h cells (Fig 2Ac & Bc). All these results suggested that the low dosage of demethylation drugs did not immediately trigger the p53 mediated cell cycle arrest in wild type cells. Previously published results [13] had demonstrated that the Decitabine treatment of wild type and p53KO MEFs caused the same degree of depletion in free DNMT1 protein activity and similar declines of genomic DNA methylation [13], which suggested that the drug inhibited an equal level of DNA demethylation in both wild type and p53KO cells. The result reported here support these previous observations [13] by showing the nucleoside analogue inhibitors such as FCDR and Decitabine are incorporated into the DNA of p53 mutant and wild type cells in equal levels. In addition p53 mutant and wild type cells replicate at equal rates during treatment with these drugs, yet p53 mutant cells eventually die while wild type cells can live.

### P53 deficient mice had shorter mean life span than control mice after lethal dosage of Decitabine injection

**Figure 3:**
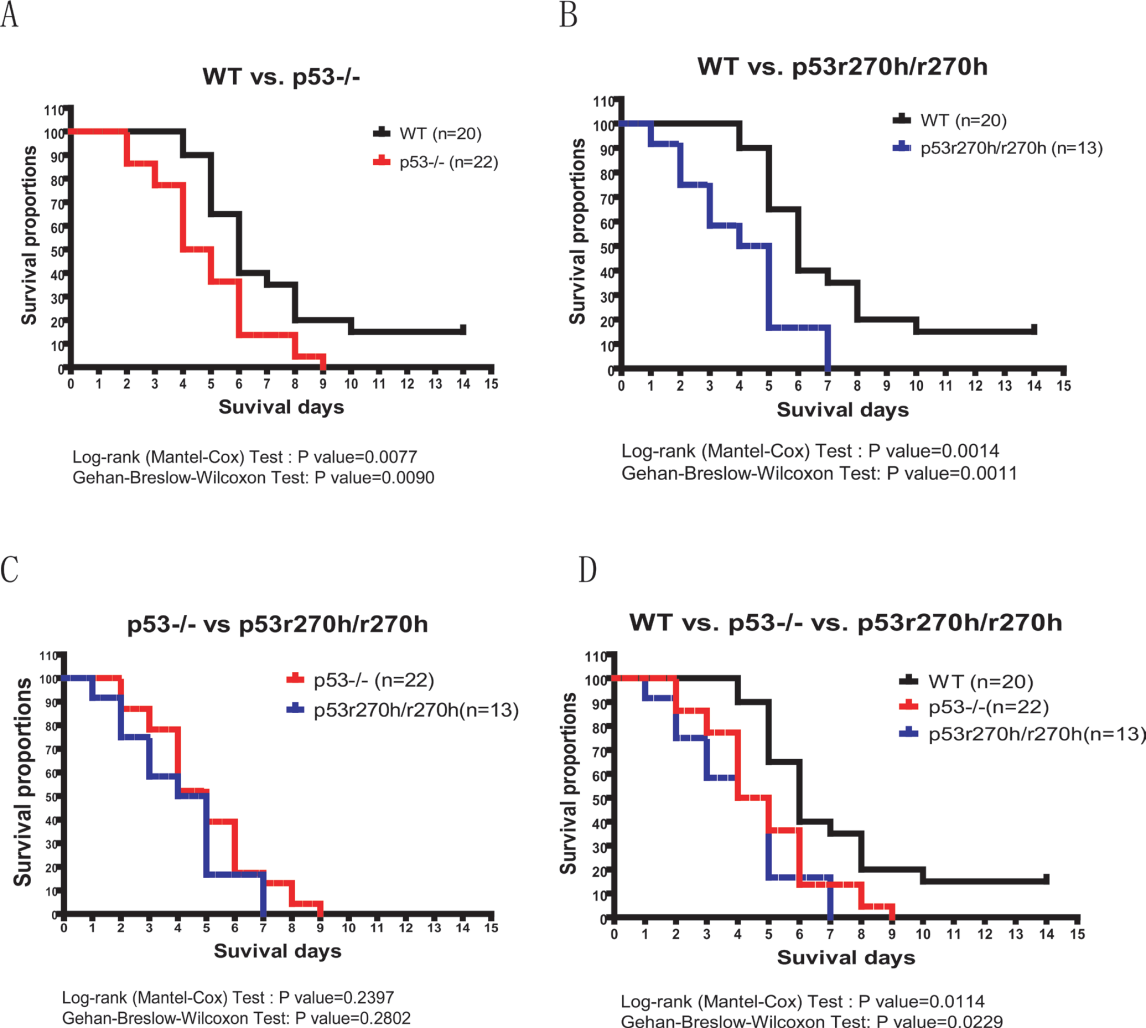
The mouse toxicity assay for p53 deficient and wildtype mice followed by Decitabine IP injections The comparison between p53KO mice and wildtype mice **(A)** or between p53r270h mice and wildtype mice **(B)** or between p53KO mice and p53r270h mice **(C)** or merged all curves **(D)**. p53KO(red), p53r270h(blue) & wildtype (black)

To study the impact of dymethylation drugs in mice we performed a toxicity assay with p53 deficient and wild type mice using a Decitabine injection. We injected lethal doses of Decitabine into mice with three different genotypes, i.e., p53 knock out, p53r270h mutation and p53 wild type. 37.5mg/kg of Decitabine was injected four times (IP) every other day into each mouse at the age of 8–12 weeks. We recorded the number of days of survival for each mouse. The day after the last injection was recorded as Day 1 for producing the survival curves. As shown in figure 3, on average, both p53 knock-out and p53r270h mice died earlier than wild type control mice. The p-values from log-rank test (p=0.0077(p53KO) and 0.0014(p53r270h)) and Gehan-Breslow-Wilcoxon tests (p =0.0090 (p53KO) and 0.0011(pp53r270h)) were both statistically significant when comparing p53 KO/mutant mice to wild type control mice. Conversely, no significant difference was found when comparing p53KO to p53r270h mice. The rapid time of death after this treatment implied of possibility of gastrointestinal toxicity of the drug. We therefore applied Hematoxylin and eosin (H&E) stain on control and drug treated intestinal tissues for three genotypes. However, our histopathological microscopy did not detect any significant changes on the intestinal tissues including mucosa, integrity of the epithelium, and the cell apoptosis (data not shown), suggesting that the mice could have died from other organ failures.

### P53 deficient human lung cancer cells were more sensitive to DNA methylation inhibitors than p53 wild type normal or cancer cells

**Figure 4:**
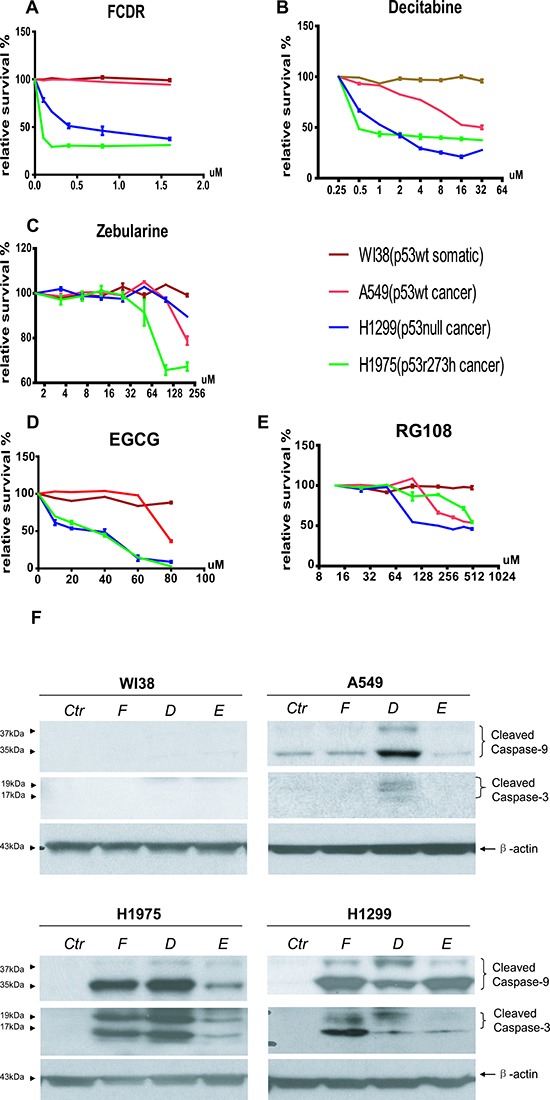
Relative cell survival plot by Guava viability assay of 5 DNMT inhibitors in human lung normal and cancer cells (**A**) FCDR, (**B**) Decitabine, (**C**) zebularine, (**D**) EGCG, (**E**) RG108. WI38(brown), A549(red), H1299(blue)& H1975(green). **(F)** Western blot of cleaved Capase3&9 in all four human lung cell lines with or with out demethylation drug treatment. Ctr=untreated control; F=FCDR; D=Decitabine; E=EGCG.

In mouse cells, we confirmed the results from previous publications that p53 deficient cells were more susceptible to DNA demethylation treatment than wild type cells, and found 4 more compounds functioning through the same pathway with a similar effect on p53 deficient cells. However all of these experiments were carried out with mouse cells and in mice. It remained unclear how human cells, especially cancer cells, would respond to these demethylation drugs. We therefore performed the guava viability-count assay for selected human lung cancer cell lines employing 5 different demethylation drug treatments. Four cell lines were included in this assay. WI38 was normal human embryonic lung fibroblast cells with wild type p53 function. A549 was human non-small cell lung carcinoma cells with wild type p53 expression. H1299 was human non-small cell lung carcinoma cells with no p53 expression (a deletion mutation). H1975 was human lung adenocarcinoma cells with homozygous missence mutated p53r273h gene. p53r273h is the human counterpart of mouse p53r270h mutation. Among the 5 drugs, FCDR, Decitabine and EGCG displayed preferential killing of the p53 null(H1299) or mutated cells (H1975) faster than p53 wild type cells and the p53 mutant cells died at lower dosages (Fig 4A, B, D). However, Zebularine and RG108, which had discriminated between p53 mutant and wild type cells in their treatment, did not show this type of specificity in the human lung cells culture (Fig 4C,E). when comparing to all the human cell lines employed, the p53 wild type normal fibroblast WI38 displayed the strongest resistance to all demethylation drug treatments. This result again confirmed that, in general, demethylation drug treatment preferentially kills p53 deficient cells in human cell culture. However, the actual efficiency of each drug may vary, depending on tissue or species specificity.

To determine the mechanism of cell death by these drugs, the activated caspase protein expression level was determined after each drug treatment. As shown in Figure 4F, FCDR Decitabine and EGCG treatment did not significantly increase cleaved Caspase 9 & 3 protein levels in p53 wild type normal WI38 cells. Similar results were also seen in p53 wild type cancer cells, A549 after FCDR and EGCG treatment. Unlike WI38, in A549, there is some background expression of cleaved caspase 9 even in the untreated control cell cultures. However, the Decitabine treatment increased the expression of cleaved caspase 9 & 3 in the A549 cells. This result is consistent with previously observation that, Decitabine treatment caused a greater cell death in A549 cells than in the WI38 cells. On the contrary, in both p53 null and mutated cells, FCDR, Decitabine and EGCG treatment induced a significantly high expression of cleaved caspase 9 & 3. This result suggested that the caspase mediated apoptosis pathway did contribute to the demethylation drugs selectively killing in the p53 deficient cells. The capase protein expression level was strongly coordinated with the growth inhibition observed in the human cell cultures even though mouse cell cultures.

### EGCG injection into nude mice significantly inhibited growth of p53 null lung Xenograft tumors but not the p53 wild type lung tumors

**Figure 5:**
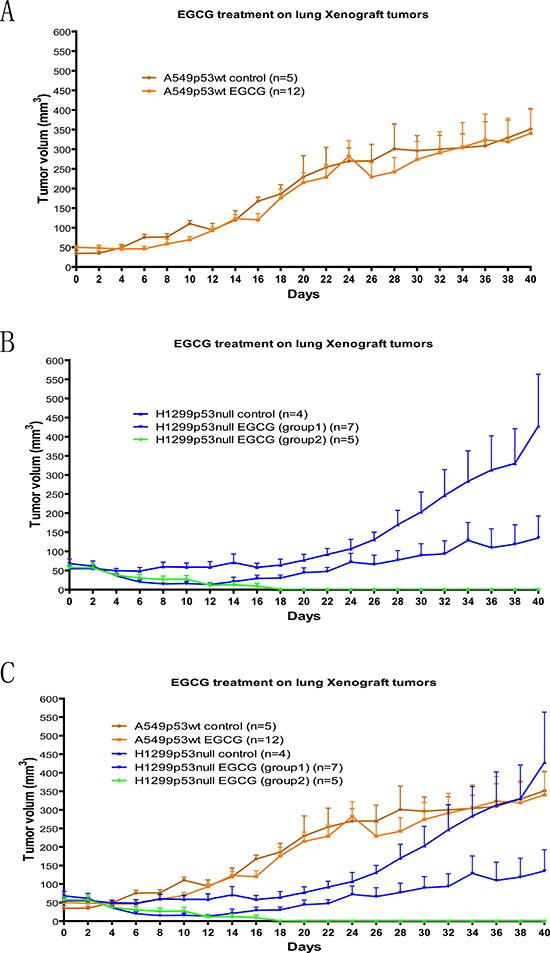
Xenografts tumor assay for EGCG affect on p53null and wildtype human lung caner cells 50mg/kg of EGCG was given by IP injection to nude (immune deficient) mice with Xenograft tumor from A549 (p53wt) and H1299 (p53null) human lung cancer cell lines. The treatment continues every other day for up to 40 days (21 injections total). The growth of p53wt tumor A549 was not significantly impacted by the EGCG injections. (**A**) A549 without EGCG injection (dark brown line) and A549 with EGCG injection (light brown line); however, the growth of p53null tumor H1299 was significantly delayed by EGCG injections. Out of 12 tested tumors, 5 of them exhibited a complete reduction of tumor volume to not being able to be detected (green line) and 7 of them exhibited a regression of tumor volume in the first 12 days and a delayed growth of the tumor after that (light blue line) comparing to H1299 without EGCG injection (dark blue line) (**B**). The comparison of all the merged curves can be seen in (**C**). p-value was obtained from repeated measure growth curve analysis. For A549 p53wt control vs. EGCG, p-value=0.58; For H1299 p53null control vs EGCG (group 1), p-value=0.003; For H1299 p53null control vs. EGCG (group 2), p-value<0.001.

Based on the cell culture result, we knew that the p53 null cancer cell H1299 was more susceptible to EGCG treatment than the p53 wild type cancer cell A549. We then investigated whether this observation would be observed in a xenograft tumor model. We injected the human lung cancer cell lines A549 and H1299 into immunocompromised nude mice to form tumor. One week later, when the tumor volume reached 50 mm^3^, 50 mg/kg of EGCG was given to these nude mice (tumor carrier) by IP injection employing a 2 day interval for up to 40 days (21 times). The tumor volume was measured on the day of each injection. We noticed that the tumor growth rates were slightly different for each cell line. For the first 20 days, the A549 control tumors (no drug used) grew faster than H1299 control tumors in nude mice. After 20 days, the H1299 control tumors grew faster. To minimize the variability in the nature growth rates of different tumor types, we only compared the results between test groups (treated with the drug) and control groups (not treated with the drug) within the same tumor type. Consistent with the results in cell culture described earlier, EGCG had no significant impact on suppressing the A549 tumor growth (Fig5A, C), but significantly delayed the H1299 tumor growth. In the first 12 days, almost all of the 12 EGCG treated H1299 tumors were reduced in size (Fig 5B, C). After 12 days, 5 out of 12 tumors had stopped growing before completely vanishing (p-value<0.001). The other 7 tumors gradually grew back in size after stopping the drug treatment but were still smaller on average than the control tumors at the same time points (p-value= 0.003). The p-values were obtained from repeated measurements of the growth curves.

## DISCUSSION

Changes in DNA methylation patterns are well known to be associated with genome wide hypomethylation and regional hypermethylation in numerous cancers [17]. These changes have been linked to both increased and decreased alterations in the transcription of both genes and non-coding RNAs considered critical to carcinogenesis. In mammals the enzymes that catalyze the transfer of methyl-groups onto cytosine residues are composed of three family members; Dnmt1, Dnmt3a and Dnmt3b. Dnmt1 methylates a cytosine residue in a CpG dinucleotide when the opposite strand base pairs GpC are methylated on the cytosine residue. It is the copy DNA methyl-transferase once the epigenetic marks are in place. Dnmt3a and 3b are de novo cytosine methyl-transferases, which lay down the initial patterns of DNA methylation.

In 2004 the Serrano group [19] first reported that the treatment of mouse embryo fibroblasts with 5-aza-2deoxycytodine, which incorporates into the DNA and blocks further DNA methylation, led to apoptosis of these cells when they were deficient in p53 but not when they had wild type p53. They demonstrated that drug treatment of these cells, with or without p53, led to the same levels of hypomethylation of these cells but that cells with wild type p53 activated the p53 protein for transcription and these cells arrested at the G2/M checkpoint where as p53 deficient cells accumulated severe chromosomal alterations and then undergo apoptosis. Thus p53 senses the levels of DNA methylation changes and after activation employs cell cycle arrest to reduce cell death.

These studies were followed up by Leonova and her colleagues [13] who found that p53 deficient cells treated with 5-aza-2-deoxycytodine, but not p53 wild type cells, induced a large increase in short-interspersed nuclear elements (SINE RNAs) and transcription of both strands of near –centromeric satellite DNAs consisting of tandem repeats and multiple species of noncoding RNAs. These are the analogous RNA species described by Haber and his colleagues in human cancers but not expressed in normal cells [12]. The absence of p53 in these cells presumably permits the epigenetic changes to occur that result in the transcription of the repetitive RNA species. Leonova went on to show that the RNA repeats induce a strong endogenous apoptosis-inducing type-1 interferon response, which kills these cells. This phenomenon was called TRAIN (transcription of repeats activates interferon).

The Serrano and Leonova results both point to a role for wild type p53 in sensing changes in epigenetic control and reducing cell death so that corrections can be made to restore proper epigenetic control. The work reported in this publication confirms and extends these observations from mouse to human cells, employing several different types of drugs that block methylation of cytosine residues, and from cell culture to mice carrying p53 deficient alleles and cancerous xenografts growing in mice. However the results reported here are not without some difficulties in the interpretation of these data. The set of drugs that block methylation of cytosine residues in DNA show some species specificities in their preferences for killing p53 deficient cells (human or mouse) and possibly even tissue or cell type differences. This may indicate off target effects of these drugs or differences in p53 functionality. Indeed the set of drugs tested here were not uniform in distinguishing mutant p53 (killed) from wild type p53 cells (survive better). The differences between these drugs will need to be explored.

The relationships between the stability of epigenetic regulation in cells and the role of p53 in that regulation, as reviewed in the introduction section of this paper, is becoming better established. Related to this is the function of p53 in normal and cancerous stem cells. Essential to understanding how wild type p53 senses epigenetic changes is the need for a mechanism that mediates the p53 response when epigenetic changes occur. One obvious mechanism comes from the observation that the p53 protein is modified by phosphorylation, acetylation, methylation, ubiquitination, sumolation and N-acetylglycosylation. Further many of these modifications result in changes in the activity and specificity of the transcriptional programs regulated by p53. Indeed some of the very same enzymatic activities that modify chromatin modify the transcription factors like p53 to coordinate the preparation of the DNA templates and the regulators of the templates. In breast and prostate cancers of humans p53 mutation gives rise to a different transcriptional program, more like embryonic stem cells [11, 21] than is observed with p53 wild type cancers.

The experiments of S. Lowe and his colleagues [22] have demonstrated that a wild type p53-mediated program of cellular senescence includes the transcription of genes involved in cytokine secretion and the attraction of phagocytic cells and NK cells that eliminate the senescent cells from the body. What is quite interesting is that the loss of p53 functions via mutation of the p53 gene results in epigenetic reprograming, the expression of repetitive satellite non-coding RNAs that also result in the production of cytokines and interferon [13]. This is a clever failsafe when a p53 mutation arises in a cell that should then result in interferon-mediated death of de novo cancer cells. Unfortunately the resultant genomic instability of cells with p53 mutations finds a path to interferon resistance and cell replication. It is of some interest that the latest immunotherapies employing antibodies to CTLA-4 and the PD-1 ligand now permit the T-cells to function and kill the cancer cells [23]. If the p53 gene is mutant and one employs low levels of 5-aza 2-deoxycytodine this will stimulate the interferon and cytokine responses and should attract T-cells and phagocytes. So it is of some interest that treatment of cancers with low levels of 5-azacytidine enhances the anti-PD-1 ligand activity after the epigenetic changes [24, 25]. Based upon this one might anticipate that both a wild type transcriptional program and a mutant p53′s failure to regulate epigenetic states in the cell, could both lead to enhanced immunotherapy when the right drugs are also employed.

It is becoming clear that the loss of the p53 protein function in cells creates a synthetic lethality and one path to exploiting this may well involve the drugs that block epigenetic fidelity. We will need to understand this path in greater detail to maximize the use of these drugs.

## MATERIALS AND METHODS

### Cell Lines and Culture Conditions

The primary mouse embryonic fibroblasts (MEFs) wt, p53−/−, p53R172H/ R172H, p53R270H/R270H was derived from C57BL/6 mice by our own lab with standard protocols. All MEF cells are cultured in Dulbecco's modified Eagle's medium (DMEM) with 10% FBS. H1299, H1975, WI38, A549 was purchased from ATCC. A549, H1299 and WI38 were cultured in DMEM with 10% FBS. H1975 was cultured in RPMI with 10% FBS.

### Chemical reagents

Zebularine, RG108 were purchased from Cayman Chemical. Decitabine,FCDR and EGCG were purchased from Sigma-Aldrich.

### Cell Proliferation Assay

Cell proliferation assay was done with Cell counting kit-SK in accordance with the manufacturer's instructions. (Dojindo Molecular Technology, Inc.). In brief, 3,000 cells per well were cultured in 96-well plate to reach the 20%–30% confluence on the second day when treated with serial dilutions of the compounds. The growth is measured by CCK-SK reagent and Victor Plate reader instrument (PerkinElmer, Waltham, MA, USA) after incubation for 5 days.

### Guava Cell Viability Assay

Guava viability assays were done in accordance with the manufacturer's instructions for Guava ViaCount (Millipore, Billerica, MA, USA). In brief, the cells (5000 cells/well, in 1 ml culture) are cultured in a 24-well plate to reach the 20%–30% confluence on the second day when treated with serial dilutions of the compounds. The growth is measured by Guava ViaCount reagent and Guava PCA instrument after incubation for 5 days.

### Mouse Experiments

Mice were housed and treated in accordance with guidelines and all the mouse experiments are done with the approval of Institutional Animal Care and Use Committee (IACUC) of the Rutgers New Jersey Medical School. Mice with p53KO/+ and p53R270H/+were purchased from The Jackson Laboratory. The nude mice NCR nu/nu were purchased from Taconic.

For the toxicity assays, 6- to 10-week-old mice were of the following genotypes, p53R270H/R270H (n = 13), p53+/+ (n = 20), and p53−/− (n = 22). Mice were treated with Decitabine (Sigma&Cayman Chemical) 37.5 mg/kg or vehicle control (1%Polyethylene glycols (PEG800) + 1% Tween 20) by intraperitoneal injection in 2 day interval for up to 7days, followed by harvesting the mouse tissues.

Xenografts tumor assays are derived from the human tumor cell lines, H1299, and A549 (4×10^6^ cells/mouse). Tumor dimensions were measured every other day, and their volumes were calculated by length (L) and width (W) by using the formula: volume =1/2 LxW^2^. Tumors were allowed to grow to 50mm^3^ prior to two-day interval of intraperitoneal administration of EGCG (Sigma) at 50mg/kg or vehicle control (1xPBS) for up to 40 days.
